# Supplementary material for: Self-selective formation of ordered 1D and 2D GaBi structures on wurtzite GaAs nanowire surfaces
Source: Nat Commun. 2021 Oct 13;12:5990. doi: 10.1038/s41467-021-26148-4 (PMC8514568; doi:10.1038/s41467-021-26148-4)
Supplement: Supplementary file 1 — Supplementary Information [file 41467_2021_26148_MOESM1_ESM.pdf]

Supplementary information for:

Self-Selective Formation of 1D and 2D GaBi Structures on Wurtzite GaAs Nanowire Surfaces

Yi Liu[1], *Johan V. Knutsson*[1], *Nate Wilson*[2], *Elliot Young*[2], *Sebastian Lehmann*[1], *Kimberly A. Dick*[3], *Chris J. Palmstrøm*[2,4], *Anders Mikkelsen*[1], *and Rainer Timm*[1*]

[1]*Department of Physics and NanoLund, Lund University, P.O. Box 118, 221 00 Lund, Sweden*

[2]*Materials Department, University of California-Santa Barbara, Santa Barbara, CA 93106, USA*

[3]*Centre for Analysis and Synthesis and NanoLund, Lund University, P.O. Box 124, 221 00 Lund, Sweden*

[4]*Department of Electrical and Computer Engineering, University of California-Santa Barbara, Santa Barbara, CA 93106, USA*

[*]e-mail: rainer.timm@sljus.lu.se

This supplementary information includes:

Supplementary Note 1: Growth of GaAs-Bi alloys and surface layers

Supplementary Fig. 1: As-grown GaAs NWs

Supplementary Fig. 2: Characterization of GaAs NWs prior to Bi deposition

Supplementary Fig. 3: Bi incorporation in $\{10\bar{1}0\}$-type facets

Supplementary Fig. 4: Autocorrelation analysis and statistics of Bi sites

Supplementary Fig. 5 and Supplementary Table 1: The ratio of single Bi sites in $\{11\bar{2}0\}$- type surfaces

Supplementary Fig. 6 and Supplementary Table 2: Detailed statistics of Bi atoms incorporated in GaAs NWs with higher step density

Supplementary Fig. 7 and Supplementary Table 3: Detailed statistics of Bi atoms incorporated in GaAs NWs with higher step density

Supplementary Note 2: Tilted appearance of $\{10\bar{1}0\}$- type facets

Supplementary Fig. 8: Wz $\{10\bar{1}0\}$-type facet with lower Bi coverage

Supplementary Fig. 9: X-ray photoelectron spectroscopy (XPS) study on NWs

Supplementary References

Supplementary Note 1

Growth of GaAs-Bi alloys and surface layers

Conventionally, Bi-based III-V compounds are synthesized by epitaxial growth using molecular beam epitaxy (MBE) onto III-V semiconductor substrates [1-3]. Incorporation of Bi atoms into As or P lattice sites through group-V exchange has been observed for epitaxial growth of diluted Bi-containing III-V alloys [4, 5]. Bi deposition onto the surface of binary III-V compounds has so far resulted in the formation of overlayers of pure Bi [6-8] or Bi-terminated reconstructions [4], depending on the deposited amount, sample annealing parameters, and surface orientation [4, 7, 9, 10], also supported by theoretical work [11, 12]. For example, (nx3)- or (nx4)-type and (2x1)-type Bi-terminated surface reconstructions have been observed on GaAs(001) and InAs(001) [4, 7, 9].

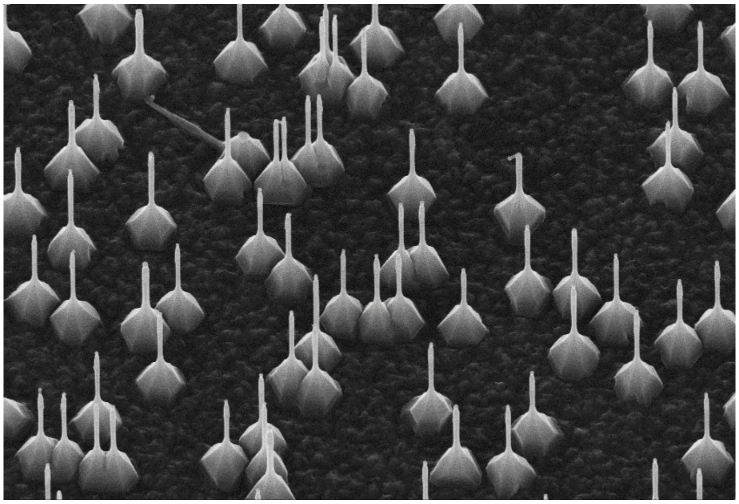

Supplementary Fig. 1 An overview scanning electron microscopy image of the as grown GaAs NWs with stems on GaAs(111)B substrates.

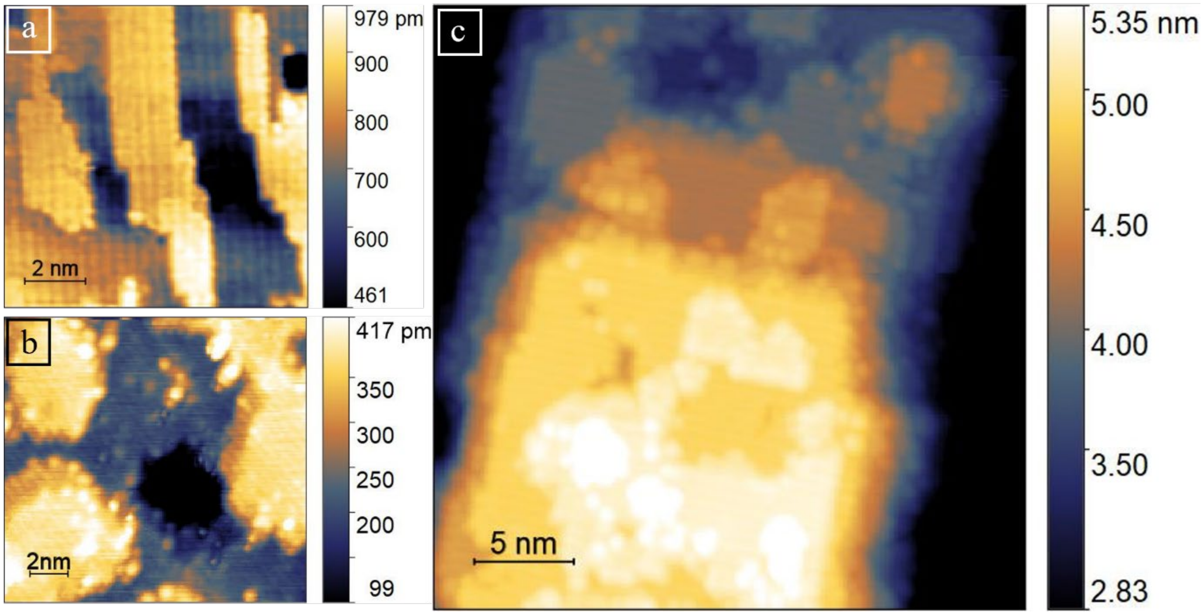

Supplementary Fig. 2 The oxide-free GaAs NWs surface after H+ cleaning. (a) $\{11\bar{2}0\}$-type facet. (b) $\{110\}$-type facet. (c) $\{10\bar{1}0\}$-type facet. Height bars are added to the right for all images. V_T = -5.1 V, I_T = 100 pA for all STM images.

The NWs consist of a Wz stem followed by a longer Zb segment with several smaller Wz segments included. In addition to the Wz and Zb segments, a thick base was formed during growth. An SEM overview image of the as-grown NWs can be seen in Supplementary Fig. 1. Upon mechanical transfer, the base remains at the growth substrate while the broken-off NWs are deposited onto the sample substrate. The most common surface terminations are $\{110\}$ or $\{111\}$A/B twin plane facets on Zb segments and the corresponding $\{11\bar{2}0\}$ or $\{10\bar{1}0\}$ facets on Wz segments, depending on specific growth and overgrowth conditions [13, 14]. After H+ cleaning, all investigated NW facets appear unreconstructed and display atomic patterns as shown in Supplementary Fig. 2, in full agreement with previous STM measurements of GaAs (and other III-V) NWs [14]. More details on these atomic arrangements can be found in previous publications [15, 16]. The nicely defect-free interface between Wz- and Zb- type facets is beneficial for directly comparing different facets while keeping the STM tip in unchanged conditions [14].

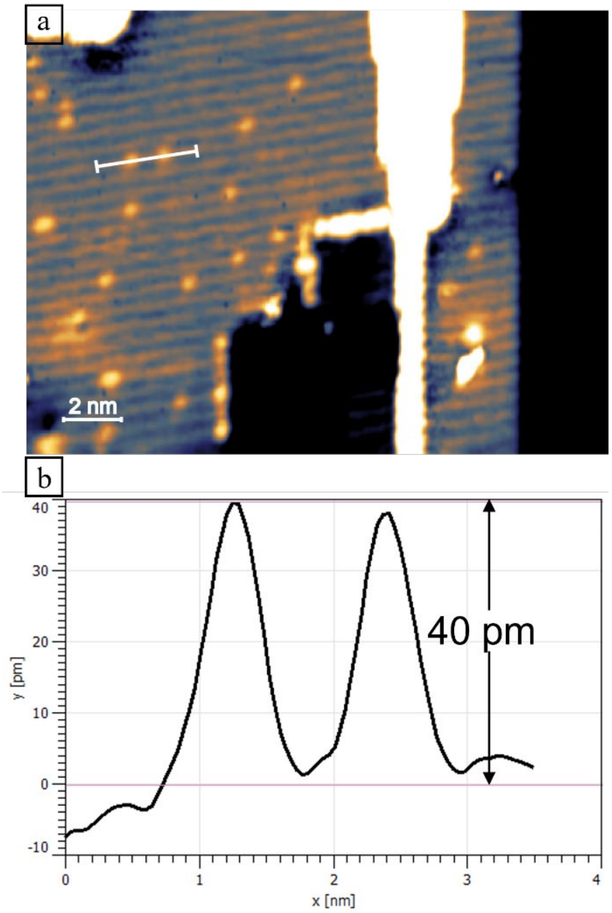

Supplementary Fig. 3 Bi incorporation in $\{10\bar{1}0\}$-type facets. (a) Atomically resolved STM image of Bi incorporation in a $\{10\bar{1}0\}$-type facet of GaAs NW surfaces. V_T = -4.4 V, I_T = 50 pA. (b) A line profile along an atomic chain including two Bi sites as marked in (a), showing a protrusion of the Bi atoms of around 40 pm.

An STM image of a $\{10\bar{1}0\}$-type facet of a GaAs NW after Bi incorporation together with a line profile across the positions of two Bi sites is shown in Supplementary Fig. 3. The line profile indicates a protrusion of the Bi atom above the surrounding GaAs surface by about 40 pm, which is close the Bi protrusion height on Wz$\{11\bar{2}0\}$- and Zb$\{111\}$-type facets (see Fig. 1e in the main manuscript).

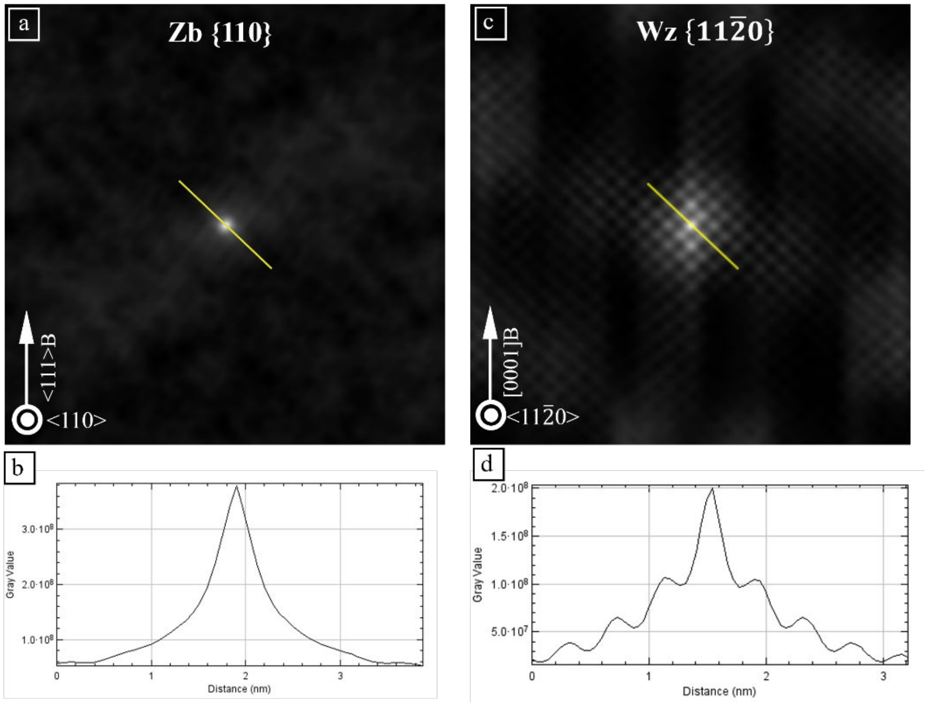

Supplementary Fig. 4 Autocorrelation analysis of Bi sites on (a) Zb{110}- and (c) Wz {11$\bar{2}$0}-type facets are shown. (a) has an area of 379 nm^2 and (c) has an area of 134 nm^2. Line profiles of the correlation pattern in one dimension, as indicated by yellow lines in (a) and (c), are shown in (b) and (d), respectively.

The meaning of an autocorrelation process is to correlate an image with itself. Autocorrelation patterns from STM images indicate the presence of short- and long-range order. To identify any ordering of incorporated Bi atoms, each individual Bi site was selected manually, then the image was converted to binary mode (containing only black and white pixels), clearly showing the Bi sites. The autocorrelation of Bi sites was obtained from a cross correlation process for the image with itself using FDmath in imageJ software, the results for Zb{110}- and Wz{11$\bar{2}$0}-type facets are shown in Supplementary Fig. 4 together with line profiles of the one-dimensional correlation pattern. On Zb{110}-type facets, no other correlation spot except for the (0,0) spot can be observed, indicating there is no position correlation of Bi sites in any direction, i.e. Bi sites are random distributed. On Wz{11$\bar{2}$0}- type facets, in contrast, a clear periodic correlation pattern can be seen. The line profile in Supplementary Fig. 4d shows that the periodicity equals the distance between atoms in a dense 2D Wz{11$\bar{2}$0}-type island, which is another strong proof that Bi atoms are located on As sites.

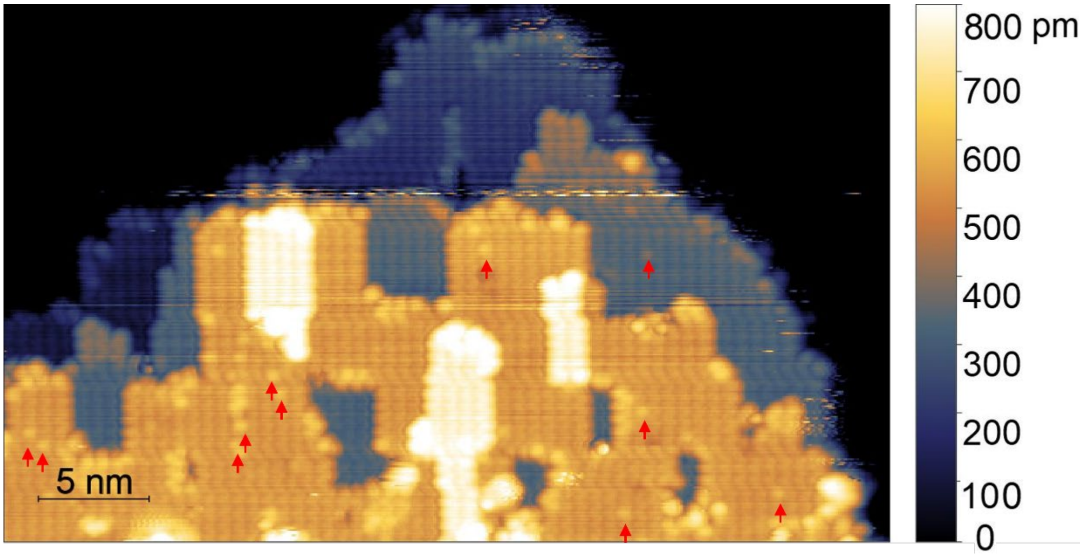

Supplementary Fig. 5 STM image of a GaAs $\{11\bar{2}0\}$-type NW facet after incorporation of Bi atoms with height bar on the right side. Single Bi sites marked with red arrows. This STM image is counted as NW 1 in Supplementary Table 1.

Nanowire	All Bi sites	Single Bi sites
1	245	10±2
2	61	4±1
3	256	8±2
In total	562	22±5
Single Bi sites ratio	(4±1)%	

Supplementary Table 1 Detailed statistics of Bi sites on $\{11\bar{2}0\}$-type facets from three different nanowires. The statistic shows only about 3-5% of the Bi atoms were found to sit individually on the $\{11\bar{2}0\}$-type facets.

A specific feature of the Bi incorporation in $\{11\bar{2}0\}$-type facets with sufficiently high step density is the presence of GaBi 2D islands and 1D chains together with the observation of only very few isolated Bi atoms. We therefore analyzed how many of all incorporated Bi atoms are positioned individually, i.e. with all nearest neighboring group-V atoms being As atoms. Such single Bi incorporation sites are highlighted in Supplementary Fig. 5, and their statistics is shown in Supplementary Table 1, which indicates that only about 3-5% of all Bi atoms sit individually in the $\{11\bar{2}0\}$-type facets. Statistical results were obtained by analyzing STM images from large areas of the NW facets across three different NWs.

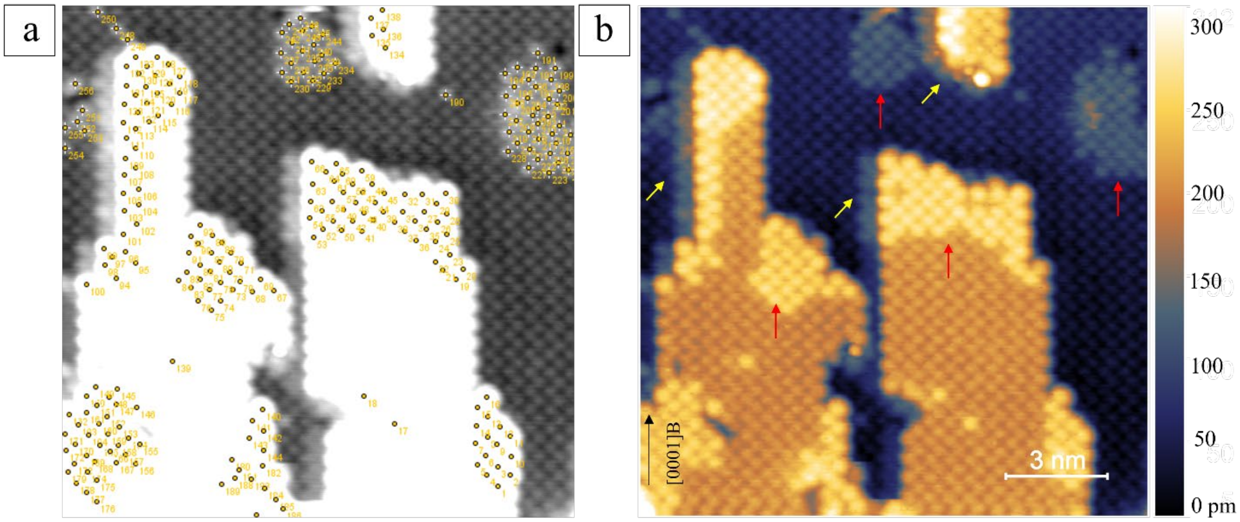

Supplementary Fig. 6 Detailed statistics of Bi atoms incorporated in GaAs NWs with higher step density (a) An STM image of a Wz $\{11\bar{2}0\}$-type facet is shown in the image, where positions of all Bi atoms are indicated and marked with counting numbers. This STM image contributes to the statistics of Bi sites density as "nanowire 3- Wz $\{11\bar{2}0\}$ facet" in Supplementary Table 2. The figure shows the same STM image as in Fig. 2d of the main manuscript, but with different contrast settings, to highlight Bi atoms in the lower atomic terrace. (b) The same STM image as in (a), but with reduced contrast, in order to visualize the atomic structures of both terraces. A height scale bar is shown. Some incorporated Bi sites are indicated with red arrows, and the yellow arrows point out some shadows of the step edges due to double tip effect.

The density of Bi sites on a NW surface imaged by STM was calculated by counting the number of Bi sites over a total area of 3.3×10^3 nm^2, 4.2×10^3 nm^2, and 1.3×10^3 nm^2 for $\{11\bar{2}0\}$-, $\{110\}$-, and $\{10\bar{1}0\}$-type facets, respectively. The Bi atoms substitution rate was calculated by dividing the Bi site density by the As atoms density, which is deducted based on the STM images as 6.2 As atoms/nm^2 for GaAs $\{11\bar{2}0\}$- and $\{110\}$- type facets and 5.2 As atoms/nm^2 for $\{10\bar{1}0\}$-type facets. One example of Bi sites counting can be seen in Supplementary Fig. 6. Detailed statistics is shown in Supplementary Table 2. Bi densities of $\rho_{\{11\bar{2}0\}}$ = 0.34±0.07 Bi atoms/nm^2, $\rho_{\{110\}}$ = 0.38±0.08 Bi atoms/nm^2, and $\rho_{\{10\bar{1}0\}}$ = 0.07±0.02 Bi atoms/nm^2 were found. That indicates that Bi in average replaces 6%, 6%, and 1% of the surface As atoms on $\{11\bar{2}0\}$-, $\{110\}$-, and $\{10\bar{1}0\}$-type facets, respectively.

Nanowire	Facet	Area (nm^2)	Number of Bi sites
1	Wz $\{11\bar{2}0\}$	968	306
2		149	43
3		225	256
4		1289	152
5		400	278
Total		3031	1035
Density of Bi sites		$0.34/nm^2$= 5.5% of surface As atoms	
1	Zb {110}	748	292
2		353	41
3		1728	879
4		1371	383
Total		4200	1595
Density of Bi sites		$0.38/nm^2$= 6.1% of surface As atoms	
6	Wz$\{10\bar{1}0\}$	427	48
7		859	36
Total		1286	84
Density of Bi sites		$0.07/nm^2$= 1.3% of surface As atoms	

Supplementary Table 2 The detailed statistics of Bi sites on the three different facets on GaAs NWs with higher step density.

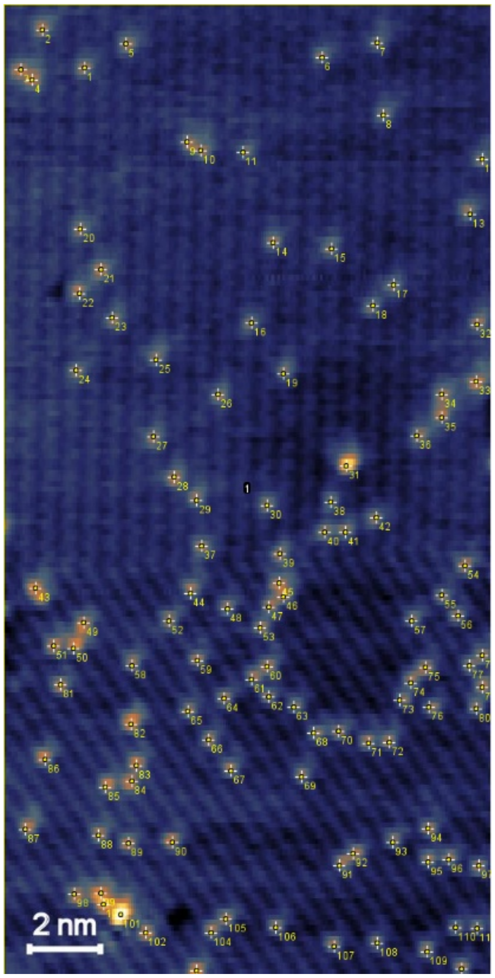

Supplementary Fig. 7 An STM image which contains both Wz $\{11\bar{2}0\}$- and Zb$\{110\}$-type facet is shown in the image, marked with the Bi counting numbers. This STM image contributes to the overall statistics of Bi sites density as “nanowire 1” in the Supplementary Table 3.

Another type of GaAs NWs was analyzed, where slightly different growth conditions resulted in larger surface terraces with a lower step density, especially on Wz $\{11\bar{2}0\}$-type facets. Supplementary Fig. 7 shows an example of Bi site counting in an STM image with an interface between $\{11\bar{2}0\}$- and $\{110\}$-type facets. Detailed statistics can be seen in Supplementary Table 3, which shows that Bi densities of $\rho_{\{11\bar{2}0\}}$ = 0.21±0.02 Bi atoms/nm^2, $\rho_{\{110\}}$ = 0.46±0.10 Bi atoms/nm^2, and $\rho_{\{10\bar{1}0\}}$ = 0.14±0.03 Bi atoms/nm^2 were found on the surfaces. This means that on average only 3.3% of the As atoms were replaced on the $\{11\bar{2}0\}$ facet, while 7.4% and 2.6% of the As atoms were substituted by Bi atoms on the $\{110\}$- and $\{10\bar{1}0\}$-type facet, respectively.

Nanowire	Facet	Area (nm^2)	Number of Bi sites
1	Wz $\{11\bar{2}0\}$	214	43
2		621	130
Total		835	173
Density of Bi sites		$0.21/nm^2$= 3.3% of surface As atoms	
1	Zb {110}	176	69
2		279	141
Total		455	210
Density of Bi sites		$0.46/nm^2$=7.4 % of surface As atoms	
3	Wz$\{10\bar{1}0\}$	600	110
4		938	100
Total		1538	210
Density of Bi sites		$0.14/nm^2$= 2.6% of surface As atoms	

Supplementary Table 3 The detailed statistics of Bi sites on the three different facets on GaAs NWs with lower step density.

The different Bi incorporation in NWs with a lower density of surface steps was also reflected by the Bi coverage on the different NW facets. The {110}- and $\{10\bar{1}0\}$-type facets have slightly more Bi sites than that on the NWs with higher step density at the same nominal amount of deposited Bi, while only 3.3% of the As atoms were replaced on the $\{11\bar{2}0\}$ facet, which is about half as many as previously. Accordingly, the probability of Bi-for-As exchange at the $\{11\bar{2}0\}$ facet drops with the decreasing density of step edges facing [0001]A/B directions, which is in agreement with the Bi incorporation model discussed in the main manuscript.

Due to radial overgrowth [13], the surface morphology (and step density) can vary slightly along a single NW. This can to some degree explain the variation in the statistics of Bi sites densities for Supplementary Table 1, 2 and 3 (i.e. the relatively large standard deviation of the values of the average Bi density). Nevertheless, the differences in the Bi site density between different NW facets are much larger than the variations within one type of surface facet, thus underlining our conclusions.

Supplementary Note 2

Tilted appearance of $\{10\bar{1}0\}$- type facets

In Fig. 3b of the main manuscript, the step edges facing <1010>-type directions are not perfectly aligned with the growth direction, but appear under some angle, which also implies that the angle between edges facing <1010>-type directions and those facing [0001]A/B directions deviates from 90°. We attribute this tilted appearance to a scanning artefact, either because the scan piezo is not perfectly aligned, or because we were scanning on a strongly inclined side facet where the projection results in some tilt (note that the $(10\bar{1}0)$ facet is 30 degrees tilted as compared to the $(11\bar{2}0)$ facet).

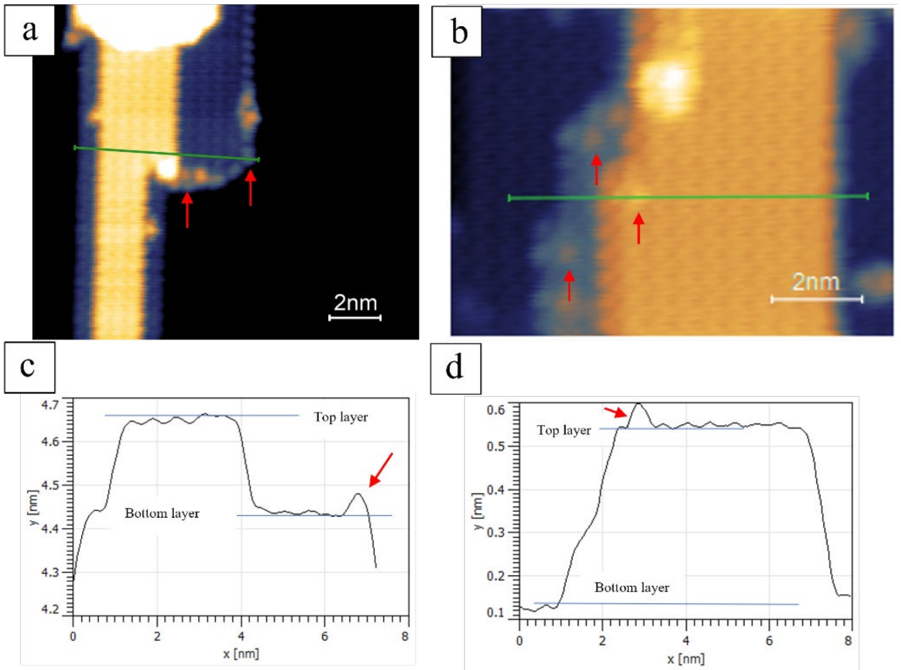

Supplementary Fig. 8 Wz $\{11\bar{2}0\}$-type facet with lower Bi coverage. (a) and (b) show STM images of Wz$\{11\bar{2}0\}$-type NW facets with around 10% (in theory) Bi coverage. (c) and (d) show the height profile of the green lines marked in (a) and (b), respectively.

In Supplementary Fig. 8a and b, STM images of Wz$\{11\bar{2}0\}$-type NW facets are shown, which have been exposed to Bi deposition for a shorter time, resulting in only about 10% (in theory) coverage. The brightness range has been adjusted so that it shows the two top terraces of GaAs: the yellow layer is sitting on top of the blue layer. A height profile is taken in Supplementary Fig. 8a and b and shown in Supplementary Fig. 8c and d, respectively. Some of the Bi sites are pointed out with red arrows, both in the STM images and in the height profile curves.

In Supplementary Fig. 8a, GaBi structures are found on the step edges facing [0001]A/B directions, similar to those seen in Fig. 2d of the main manuscript, which shows the initial state of Bi incorporation. Due to the much lower Bi coverage, the structure size is by far too small to be called a 2D GaBi nanostructure. In Supplementary Fig. 8b, there are some small GaBi structures containing 3-5 Bi atomic sites scattered on the surface, however, not connected yet. These results are also in agreement with the Bi incorporation mechanism discussed in the main manuscript.

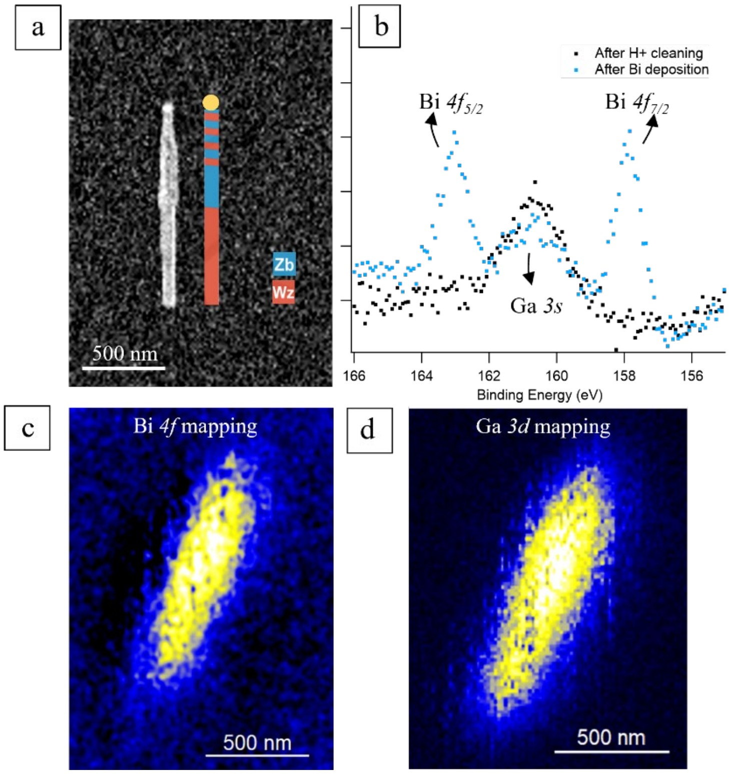

Supplementary Fig. 9 X-ray photoelectron spectroscopy (XPS) study on NW. (a) shows a scanning electron microscopy image of a typical GaAs NW. (b) presents XPS Bi *4f* core level spectra before and after Bi incorporation, measured in the middle of the same NW shown in a. (c) and (d) show 2D SPEM images of the same NW studied in (a) and (b), illustrating the distribution of (c) Bi 4f and (d) Ga *3d* signals. In both images, the distribution of the Bi and Ga signal, respectively, is in good agreement with the shape of the NW in (a).

We have performed XPS with an X-ray beam size of about 120 nm at the ESCA Microscopy beamline of the Elettra synchrotron facility in Trieste, Italy, providing nanofocus XPS and scanning photoelectron microscopy (SPEM) [17, 18]. GaAs NWs were mechanically transferred from their growth substrate onto Si substrates containing metal markers, so that the same NW could be located in SPEM images before and after Bi deposition. Supplementary Fig. 9a shows a scanning electron microscopy image of a specific NW together with its structural model, containing both Wz and Zb segments. Supplementary Fig. 9b shows XPS spectra of the Bi *4f* core-level, acquired in the middle of the NW shown in Supplementary Fig. 9a, before and after Bi deposition. We can see a distinct Bi *4f* doublet after Bi deposition (the blue spectra), obtained from the NW. The XPS intensity is relatively low because the signal only comes from one NW with about half a monolayer of Bi on the surface. We also performed 2D SPEM mapping of Bi *4f* and Ga *3d* core-levels on the same NW, which are shown in Supplementary Fig. 9c and d, respectively. The color scale corresponds to the distribution of the Bi and Ga signal in 2D. We can see that both images show the contrast in the same shape and

size as the NW. Supplementary Fig. 9c indicates that the majority of the Bi *4f* signal comes from the NW and that there is almost no Bi found on the Si substrate. This agrees with the STM results showing that Bi atoms have incorporated into the NW surface and formed chemical bonds, as we illustrated in the main manuscript, while most of the Bi atoms that have been evaporated onto the Si substrate simultaneously seem to have desorbed again. Considering the structure of the NW, with large Wz segments, and the STM images shown in Figs. 1-3 of the main manuscript, we attribute the Bi signal to mainly arise from Bi atoms incorporated in the GaAs III-V lattice of the NW surface.

Supplementary References

1. Partin, D.L., et al., *Growth and characterization of epitaxial bismuth films.* Physical Review B, 1988. **38**(6): p. 3818-3824.
2. Kim, Y., et al., *Growth habit of rhombohedral Bi thin films on zinc-blende CdTe substrates with various orientations.* Journal of Vacuum Science & Technology A: Vacuum, Surfaces, and Films, 1999. **17**(6): p. 3473-3476.
3. Tadaaki, N., et al., *Epitaxial Growth of Single-Crystal Ultrathin Films of Bismuth on Si(111).* Japanese Journal of Applied Physics, 2000. **39**(7S): p. 4567.
4. Duzik, A.J. *Bismuth-induced surface structure and morphology in III-V semiconductors*. in *Nanosensors, Biosensors, and Info-Tech Sensors and Systems 2015*. 2015. International Society for Optics and Photonics.
5. Krammel, C.M., et al., *Incorporation of Bi atoms in InP studied at the atomic scale by cross-sectional scanning tunneling microscopy.* Physical Review Materials, 2017. **1**(3): p. 034606.
6. AlZahrani, A.Z. and G.P. Srivastava, *Density-functional calculations for self-assembled Bi-nanolines on the InAs(100) surface.* Journal of Applied Physics, 2009. **106**(5): p. 053713.
7. Ahola-Tuomi, M., et al., *Formation of an ordered pattern of Bi nanolines on InAs(100) by self-assembly.* Applied Physics Letters, 2008. **92**(1): p. 011926.
8. Ludeke, R., et al., *Structural and electronic properties of Bi/GaAs(110).* Journal of Vacuum Science & Technology B: Microelectronics Processing and Phenomena, 1989. **7**(4): p. 936-944.
9. Bastiman, F., et al., *Bi incorporation in GaAs(100)-2×1 and 4×3 reconstructions investigated by RHEED and STM.* Journal of Crystal Growth, 2012. **341**(1): p. 19-23.
10. McGinley, C., et al., *The interaction of bismuth with the GaAs (111) B surface.* Applied surface science, 1999. **152**(3-4): p. 169-176.
11. Laukkanen, P., et al., *Bismuth-containing c(4×4) surface structure of the GaAs(100) studied by synchrotron-radiation photoelectron spectroscopy and ab initio calculations.* Journal of Electron Spectroscopy and Related Phenomena, 2014. **193**: p. 34-38.
12. Duzik, A., et al., *Surface reconstruction stability and configurational disorder on Bi-terminated GaAs(001).* Physical Review B, 2013. **87**(3).
13. Knutsson, J.V., et al., *Atomic Scale Surface Structure and Morphology of InAs Nanowire Crystal Superlattices: The Effect of Epitaxial Overgrowth.* ACS Applied Materials & Interfaces, 2015.
14. Hjort, M., et al., *Direct Imaging of Atomic Scale Structure and Electronic Properties of GaAs Wurtzite and Zinc Blende Nanowire Surfaces.* Nano Letters, 2013. **13**(9): p. 4492-4498.
15. Hjort, M., et al., *Crystal Structure Induced Preferential Surface Alloying of Sb on Wurtzite/Zinc Blende GaAs Nanowires.* Nano Letters, 2017. **17**(6): p. 3634-3640.
16. Hjort, M., et al., *Electronic and Structural Differences between Wurtzite and Zinc Blende InAs Nanowire Surfaces: Experiment and Theory.* ACS Nano, 2014. **8**(12): p. 12346-12355.
17. Amati, M., et al., *Photoelectron microscopy at Elettra: Recent advances and perspectives.* Journal of Electron Spectroscopy and Related Phenomena, 2018. **224**: p. 59-67.
18. McKibbin, S.R., et al., *Operando Surface Characterization of InP Nanowire p–n Junctions.* Nano Letters, 2020. **20**(2): p. 887-895.
